# Supplementary material for: Novel Driver Strength Index highlights important cancer genes in TCGA PanCanAtlas patients
Source: PeerJ. 2022 Aug 11;10:e13860. doi: 10.7717/peerj.13860 (PMC9375969; doi:10.7717/peerj.13860)
Supplement: Supplemental Information 2 [file peerj-10-13860-s002.zip › COHORTS/genes plots/cohorts/2021_8_16_14_9_distribution_cohorts_females_STAD.pdf]

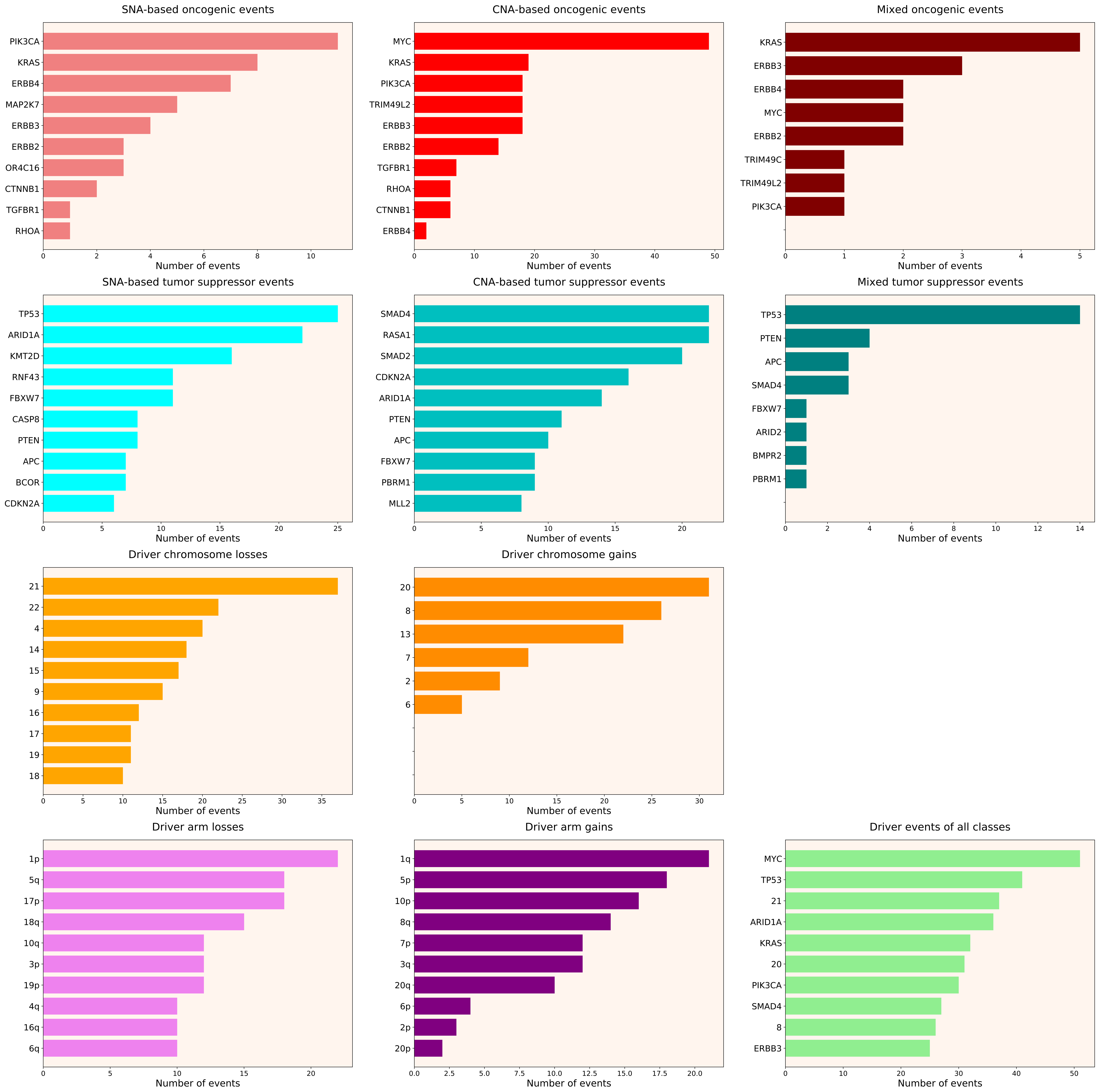

SNA-based oncogenic events
PIK3CA
KRAS
ERBB4
MAP2K7
ERBB3
ERBB2
OR4C16
CTNNB1
TGFBR1
RHOA
0 2 4 6 8 10
Number of events
CNA-based oncogenic events
MYC
KRAS
PIK3CA
TRIM49L2
ERBB3
ERBB2
TGFBR1
RHOA
CTNNB1
ERBB4
0 10 20 30 40 50
Number of events
Mixed oncogenic events
KRAS
ERBB3
ERBB4
MYC
ERBB2
TRIM49C
TRIM49L2
PIK3CA
0 1 2 3 4 5
Number of events
SNA-based tumor suppressor events
TP53
ARID1A
KMT2D
RNF43
FBXW7
CASP8
PTEN
APC
BCOR
CDKN2A
0 5 10 15 20 25
Number of events
CNA-based tumor suppressor events
SMAD4
RASA1
SMAD2
CDKN2A
ARID1A
PTEN
APC
FBXW7
PBRM1
MLL2
0 5 10 15 20
Number of events
Mixed tumor suppressor events
TP53
PTEN
APC
SMAD4
FBXW7
ARID2
BMPR2
PBRM1
0 2 4 6 8 10 12 14
Number of events
Driver chromosome losses
21
22
4
14
15
9
16
17
19
18
0 5 10 15 20 25 30 35
Number of events
Driver chromosome gains
20
8
13
7
2
6
0 5 10 15 20 25 30
Number of events
Driver arm losses
1p
5q
17p
18q
10q
3p
19p
4q
16q
6q
0 5 10 15 20
Number of events
Driver arm gains
1q
5p
10p
8q
7p
3q
20q
6p
2p
20p
0.0 2.5 5.0 7.5 10.0 12.5 15.0 17.5 20.0
Number of events
Driver events of all classes
MYC
TP53
21
ARID1A
KRAS
20
PIK3CA
SMAD4
8
ERBB3
0 10 20 30 40 50
Number of events
